# Linear epitopes of SARS-CoV-2 spike protein elicit neutralizing antibodies in COVID-19 patients

**DOI:** 10.1038/s41423-020-00523-5

**Published:** 2020-09-07

**Authors:** Yang Li, Dan-yun Lai, Hai-nan Zhang, He-wei Jiang, Xiaolong Tian, Ming-liang Ma, Huan Qi, Qing-feng Meng, Shu-juan Guo, Yanling Wu, Wei Wang, Xiao Yang, Da-wei Shi, Jun-biao Dai, Tianlei Ying, Jie Zhou, Sheng-ce Tao

**Affiliations:** 1 Shanghai Center for Systems Biomedicine, Key Laboratory of Systems Biomedicine (Ministry of Education), Shanghai Jiao Tong University, 200240 Shanghai, China; 2 MOE/NHC/CAMS Key Laboratory of Medical Molecular Virology, School of Basic Medical Sciences, Shanghai Medical College, Fudan University, 200032 Shanghai, China; 3 Foshan Fourth People’s Hospital, 528000 Foshan, China; 4 Key Laboratory of RNA Biology, Institute of Biophysics, Chinese Academy of Sciences, 100101 Beijing, China; 5 National Institutes for Food and Drug Control, Tiantan Xili #2, Beijing, China; 6 CAS Key Laboratory of Quantitative Engineering Biology, Guangdong Provincial Key Laboratory of Synthetic Genomics and Shenzhen Key Laboratory of Synthetic Genomics, Shenzhen Institute of Synthetic Biology, Shenzhen Institutes of Advanced Technology, Chinese Academy of Sciences, Shenzhen, China

**Keywords:** Immunogenetics, Viral infection

COVID-19 is caused by SARS-CoV-2.^1,2^ By July 25, 2020, globally, 15,672,841 diagnosed cases and 638,352 deaths were reported (https://coronavirus.jhu.edu/map.html).^3^ High titers of Spike protein (S protein)-specific antibodies are found in the blood of COVID-19 patients, especially IgG for both SARS-CoV^4^ and SARS-CoV-2.^5,6^ Because of the central role that S protein plays in the entry of the virus into the host cell, S1 and, more specifically, the RBD (receptor-binding domain) is the most targeted region for the development of COVID-19 therapeutic antibodies^7,8^ and vaccines.^9^ It is known that in addition to the RBD, other areas/epitopes of S protein may also elicit neutralizing antibodies.^10^ However, antibody responses to full-length S protein have not been investigated at epitope resolution, and the capability of linear epitopes to elicit neutralizing antibodies has still not been explored.

To precisely decipher the B-cell linear epitopes of the S protein, we constructed a peptide microarray. A total of 211 peptides (Supplementary Table 1) were synthesized and conjugated to BSA (Supplementary Fig. 1a–c). The conjugates along with control proteins were prepared in triplicate at three dilutions. High reproducibility among triplicate spots or repeated arrays for serum profiling was achieved (Supplementary Fig. 1d, e). Peptides with variable concentrations may enable dynamic detection of antibody responses and indicate that antibodies against different epitopes may have different kinetic characteristics (Supplementary Figs. 1f and 2a). Moreover, an inhibitory assay using free peptides verified the specificity of the signals generated against the peptides (Supplementary Fig. 2b).

Fifty-five sera from convalescent COVID-19 patients and 18 control sera (Supplementary Table 2) were screened on the peptide microarray for both IgG and IgM responses (Fig. 1a and Supplementary Fig. 3). For IgG, COVID-19 patients were completely separated from controls, and distinct and specific signals were shown for some peptides. In contrast, the assay results were not distinct enough for IgM responses. We then focused on IgG for further analysis. Epitope maps of S protein were generated based on the response frequency (Fig. 1b).

**Fig. 1:**
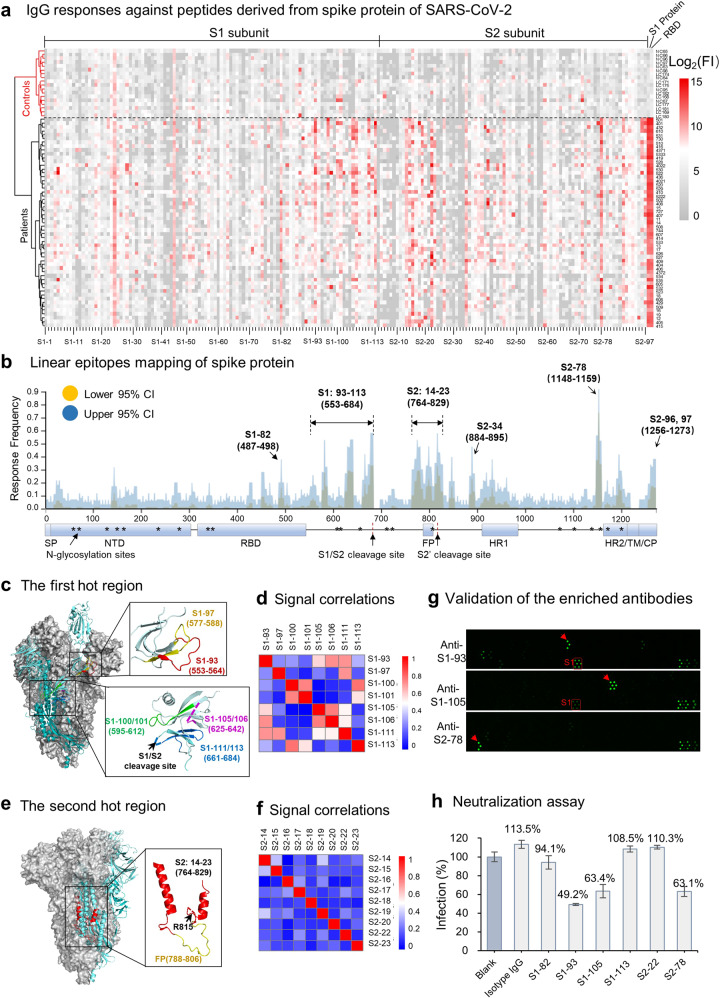
Linear epitope mapping of SARS-CoV-2 S protein and neutralizing activities of the elicited antibodies. **a** Heatmap of IgG antibody responses of 55 sera from COVID-19 convalescent patients and controls (healthy donors and lung cancer patients). FI fluorescence intensity. **b** Epitope mapping according to the response frequency. CI confidence interval. **c** Detailed structural information of the epitopes of the first hot areas on S protein (PDB: 6VYB). **d** Correlations of the antibody responses among the peptides for the first hot areas. **e** Detailed structural information of the epitopes of the second hot areas on S protein (PDB: 6VYB). **f** Correlations of the antibody responses among the peptides for the second hot areas. **g** Peptide microarray results for the enriched epitope-specific antibodies. **h** Neutralization assay with epitope-specific antibodies. Infection rates for each sample relative to that of the blank control are indicated. Triplicate experiments were performed, and the error bars indicate the SEM (standard error of the mean) value

Primarily, there are three hot epitope areas across S protein. The first is on the CTD (C terminal domain) that immediately follows the RBD, i.e., from S1–93 to S1–113. Interestingly, the identified epitopes, S1–93, 97, 100/101, 105/106, 111, and 113, are located predominantly in flexible loops (Fig. 1c). In addition, the signals of some epitopes had moderate correlations with others (Fig. 1d), and most of these epitopes were positively correlated with S1 (Supplementary Fig. 4c–f). The second hot area is from S2–14 to S2–23, including the FP (fusion peptide, aa 788–806) region and the S2′ cleavage site (R815) (Fig. 1e). In contrast to those for the first hot region, the antibody responses against epitopes of this region had poor correlations with each other (Fig. 1f), possibly due to the capability of this region to generate continuous but competitive epitopes. Moreover, part of this area is shielded by other parts in trimeric S (Fig. 1e), suggesting that this part would be easily accessed by the immune system after the departure of S2 from S1. The third hot area is S2–78 or aa 1148–1159, connecting HR1 (heptad repeat 1) and HR2 (heptad repeat 2) on the S2 subunit. IgG antibodies against this epitope can be detected in ~90% of COVID-19 patients, indicating that it is an extremely dominant epitope. In addition to these three areas, S2–34 (aa 884–895) and S2–96/97 (aa 1256–1273) also elicited antibodies in some patients.

The RBD can elicit a high titer of antibodies and highly correlates with the S1 protein^6^ (Supplementary Fig. 4a), suggesting that the RBD is a dominant region. A peptide, S1–82, located exactly on the surface of the RBM (receptor-binding motif) (Supplementary Fig. 5a), was identified as an epitope. However, further analysis demonstrated that the epitope had poor specificity (Supplementary Fig. 5b), probably due to sequence similarity (Supplementary Fig. 5c–g). Thus, the validity of epitope S1–82 may need further investigation. Other than S1–82, no significant binding was observed for the rest of the peptides located at the RBD, suggesting that conformational epitopes are dominant for the RBD.

In addition to the RBD, other areas/epitopes of S protein may also elicit neutralizing antibodies.^10^ To explore this possibility, we chose six representative epitopes, i.e., S1–82, S1–93, S1–105, S1–113, S2–22, and S2–78, to test. Antibodies that specifically bind these epitopes were separately enriched from five sera. High specificity of these antibodies, except for S1–82, was demonstrated (Fig. 1g, Supplementary Fig. 6). A pseudotype virus neutralization assay with the enriched antibodies was then performed. Because of the limited amount of the enriched epitope-specific antibodies, the assay was performed for only a single point with the highest antibody concentration that was applicable for each epitope-specific antibody. Surprisingly, the antibodies against three epitopes, i.e., S1–93, S1–105, and S2–78, exhibited potent neutralizing activity, with virus infection inhibitory efficiencies of 51%, 35%, and 35% at 8.3, 10.4, and 21 μg/mL, respectively (Fig. 1h). However, S1–82, S1–113, and S2–22 at 2.6, 7.6, and 21 μg/mL, respectively, were not effective.

S1–93 is located at the CTD of S1. The antibody against this epitope has neutralizing activity, which is consistent with a recent study.^11^ Antibody binding to this epitope may affect the conformational change of S for ACE2 binding. S1–105 also belongs to the CTD but is close to the S1/S2 cleavage site. The antibodies may block effective protease cleavage at this site, which is critical for the entry of the virus. S2–78 is located adjacent to HR2, and antibodies that bind to this epitope may interfere with the formation of 6-HB (helical bundle), an essential structure for cell membrane fusion.^12^

Potent neutralizing antibodies could provide therapeutic and prophylactic reagents to fight against the COVID-19 pandemic.^13^ However, it is risky to focus only on the RBD region, and evolutionary pressure on this “hot” area may cause potential mutations in this region. This may overcome or diminish the effectiveness of the RBD region-centered therapeutic antibodies and vaccines in the development pipeline.^14^ Thus, identification of other domains or epitopes that can elicit neutralizing antibodies is essential as well. The combination of the potent antigenicity of these peptides and neutralizing activity of the corresponding antibodies makes these epitopes potential candidates for both therapeutic antibodies and vaccine development.

There are some limitations in this study. Because a very limited amount of purified antibodies could be enriched from valuable serum samples, we could not perform a full set of neutralization assays by serial dilution to obtain the exact IC_50_ values. The IC_50_ values estimated based on the present data were 5–20 μg/mL for the antibodies. However, monoclonal antibodies presumably have better neutralization activity, so it is an option to acquire reactive B-cell clones and express recombinant monoclonal antibodies.^2,7^ Because of the poor availability of convalescent sera, we did not thoroughly examine all the peptides that may potentially raise neutralizing antibodies. The other epitopes that raised antibodies may also have neutralizing activities, such as S1–97, which is very close to S1–93, or the peptides derived from the FP region, such as S2–18, –19, and –20, which are worth further investigation.

## Supplementary information

Supplementary information

## Data Availability

The peptide microarray data are deposited in the Protein Microarray Database (http://www.proteinmicroarray.cn) under the accession number PMDE242. Additional data related to this paper may be requested from the authors.
