# Supplementary material for: Linear epitopes of SARS-CoV-2 spike protein elicit neutralizing antibodies in COVID-19 patients
Source: Cell Mol Immunol. 2020 Sep 7;17(10):1095–7. doi: 10.1038/s41423-020-00523-5 (PMC7475724; doi:10.1038/s41423-020-00523-5)
Supplement: Supplementary file 1 — Supplementary information [file 41423_2020_523_MOESM1_ESM.pdf]

Supplementary Information

Linear epitopes of SARS-CoV-2 spike protein elicit neutralizing antibodies in COVID-19 patients

Li, et al

Table of contents:

Materials and Methods
Supplementary Fig. 1. Peptide design and microarray fabrication.
Supplementary Fig. 2. Detection of the SARS-CoV-2 specific antibody responses by using the peptide microarray.
Supplementary Fig. 3. IgM responses against peptides derived from S protein.
Supplementary Fig.4. Correlation analysis among antibody responses against S1 subunit derived epitopes and S1 protein.
Supplementary Fig.5. Cross-activity of anti-S2-82 antibodies.
Supplementary Fig.6. Epitope-specific antibody depletion from sera.
Supplementary Table 1 The peptides synthesized in this study
Supplementary Table 2 Serum samples used in this study

Materials and Methods

Peptide synthesis and conjugation with BSA

The N-terminal amidated peptides were synthesized by GL Biochem, Ltd. (Shanghai, China). Each peptide was individually conjugated with BSA using Sulfo-SMCC (Thermo Fisher Scientific, MA, USA) according to the manufacture's instruction. Briefly, BSA was activated by Sulfo-SMCC in a molar ratio of 1: 30, followed by dialysis in PBS buffer. The peptide with cysteine was added in a w/w ratio of 1:1 and incubated for 2 h, followed by dialysis in PBS to remove free peptides. A few conjugates were randomly selected for examination by SDS-PAGE. For the conjugates of biotin-BSA-peptide, before conjugation, BSA was labelled with biotin by using NHS-LC-Biotin reagent (Thermo Fisher Scientific, MA, USA) with a molar ratio of 1: 5, and then activated by Sulfo-SMCC.

Peptide microarray fabrication

The peptide-BSA conjugates as well as S1 protein, RBD protein and N protein of SARS-CoV-2, along with the negative (BSA) and positive controls (anti-Human IgG and IgM antibody), were printed in triplicate on PATH substrate slide (Grace Bio-Labs, Oregon, USA) to generate identical arrays in a 1 x 7 subarray format using Super Marathon printer (Arrayjet, UK). The microarrays were stored at -80°C until use.

Patients and samples

The Institutional Ethics Review Committee of Foshan Fourth Hospital, Foshan, China approved this study and the written informed consent was obtained from each patient.

COVID-19 patients were hospitalized and received treatment in Foshan Forth hospital during the period from 2020-1-25 to 2020-3-8 with variable stay time (**Supplementary Table 2**). Serum from each patient was collected on the day of hospital discharge when the standard criteria were met according to Diagnosis and Treatment Protocol for Novel Coronavirus Pneumonia (Trial Version 5), released by the National Health Commission & State Administration of Traditional Chinese Medicine. The basic criteria are the same with that in the Diagnosis and Treatment Protocol for Novel Coronavirus Pneumonia (Trial Version 7)[1]. Briefly, the key points of the discharge criteria are: 1) Body temperature is back to normal for more than three days; 2) Respiratory symptoms improve obviously; 3) Pulmonary imaging shows obvious absorption of inflammation; 4) Nuclei acid tests negative twice consecutively on respiratory tract samples such as sputum and nasopharyngeal swabs (sampling interval being at least 24 hours). From 2020-2-28, standard criteria of discharge were modified by adding one item that nuclei acid tests should be negative on anal swab sample. Sera of the control group from Lung cancer patients and healthy donors were collected from Ruijin Hospital, Shanghai, China. All the sera were inactivated at 56 °C for 30 min and stored at -80°C until use.

Microarray-based serum analysis

A 7-chamber rubber gasket was mounted onto each slide to create individual chambers for the 7 identical subarrays. The microarray was used for serum profiling as described previously with minor modifications[2]. Briefly, the arrays stored at -80°C were warmed to room temperature and then incubated in blocking buffer (3% BSA in 1×PBS buffer with 0.1% Tween 20) for 3 h. A total of 400 μL of diluted sera or antibodies was incubated with each subarray for 2 h. The sera were diluted at 1:200 for most samples and for competition experiment, free peptides were added at a concentration of 0.25 mg/mL. For the enriched antibodies, 0.1-0.5 μg antibodies were included in 400 μL

incubation buffer. The arrays were washed with 1×PBST and bound antibodies were detected by incubating with Cy3-conjugated goat anti-human IgG and Alexa Fluor 647-conjugated donkey anti-human IgM (Jackson ImmunoResearch, PA, USA), which were diluted for 1: 1,000 in 1×PBST. The incubation was carried out at room temperature for 1 h. The microarrays were then washed with 1×PBST and dried by centrifugation at room temperature and scanned by LuxScan 10K-A (CapitalBio Corporation, Beijing, China) with the parameters set as 95% laser power/ PMT 550 and 95% laser power/ PMT 480 for IgM and IgG, respectively. The fluorescent intensity was extracted by GenePix Pro 6.0 software (Molecular Devices, CA, USA).

Purification of epitope-specific antibodies

Depends on the availability，200-500 μL serum from COVID-19 convalescent patient was two-fold diluted in 1×PBS and then pre-incubated with streptavidin beads to eliminate non-specific binding. For each epitope, 100 μg peptides conjugated with biotin-BSA were coated to 100 μL streptavidin magnetic beads (Invitrogen, MA, USA) in 1×PBS buffer at room temperature for 1 h. The protein-coated streptavidin beads were washed 4 times in 1×PBS containing 0.1% BSA, and incubated with the pre-cleaned serum in 1×PBS at 4°C for 4 h. The streptavidin beads were then washed 3 times in 1×PBS containing 0.1% BSA, and eluted with 0.2 M glycine, 1 mM EGTA, pH 2.2. Finally, the antibodies were neutralized with 1M Tris-HCl, pH8.0. The concentration of the purified antibody was monitored by silver staining.

Pseudotyped Virus Neutralization

The neutralization assay was performed as described[3]. Briefly, 293 T cells were co-transfected with expression vectors of pcDNA3.1-SARS-CoV-2-S (encoding SARS-

CoV-2 S protein) and pNL4-3.luc.RE bearing the luciferase reporter-expressing HIV-1 backbone. The supernatants containing SARS-CoV-2 pseudotyped virus were collected 48 h post-transfection. Antibodies or isotype IgG control (Thermo Fisher Scientific, MA, USA) in DMEM supplemented with 10% fetal calf serum were incubated with pseudoviruses at 37°C for 1 h and then the mixtures were added to monolayer Huh-7 cells (10^4 per well in 96-well plates). Twelve h after infection, culture medium was refreshed and then the cells were incubated for an additional 48 h. The luciferase activity was calculated for the detection of relative light units using the Bright-Glo Luciferase Assay System (Promega, WI, USA). Huh-7 cells were subsequently lysed with 50 μl lysis reagent (Promega, WI，USA), and 30 μl of the lysates were transferred to 96-well Costar flat-bottom luminometer plates (Corning Costar, MA, USA) for the detection of relative light units using the Firefly Luciferase Assay Kit (Promega, WI, USA) and an Ultra 384 luminometer (Tecan, Switzerland).

Data analysis and software

Signal Intensity was defined as the median of the foreground subtracted by the median of background for each spot and then averaged the triplicate spots for each peptide or protein. IgG and IgM data were analyzed separately. Pearson correlation coefficient between two proteins or indicators and the corresponding *p* value was calculated by SPSS software under the default parameters. Cluster analysis was performed by pheatmap package in R[4]. To calculate the response frequency of each epitope specific

antibody, mean signal + 3*SD of the control sera were used to set the threshold. The epitope map was generated by ImmunomeBrowser issued by IEDB (Epitope Prediction and Analysis Tools)[5]. Visualization of the structural details were processed by Pymol (https://pymol.org/2/).

References

1. National Health Commission & National Administration of Traditional Chinese Medicine. Diagnosis and Treatment Protocol for Novel Coronavirus Pneumonia (Trial Version 7). *Chinese medical journal* **133**, 1087–1095 (2020).
2. Li, Y. *et al.* Longitudinal serum autoantibody repertoire profiling identifies surgery-associated biomarkers in lung adenocarcinoma. *EBioMedicine* **53**, 102674 (2020).
3. Wu, Y. et al. Identification of Human Single-Domain Antibodies against SARS-CoV-2. *Cell Host & Microbe* (2020). doi:10.1016/j.chom.2020.04.023.
4. R, K. Pheatmap: Pretty Heatmaps. https://cran.r-project.org/web/packages/pheatmap/index.html (2015).
5. Dhanda, S. K. *et al.* ImmunomeBrowser: A tool to aggregate and visualize complex and heterogeneous epitopes in reference proteins. *Bioinformatics* **34**, 3931–3933 (2018).

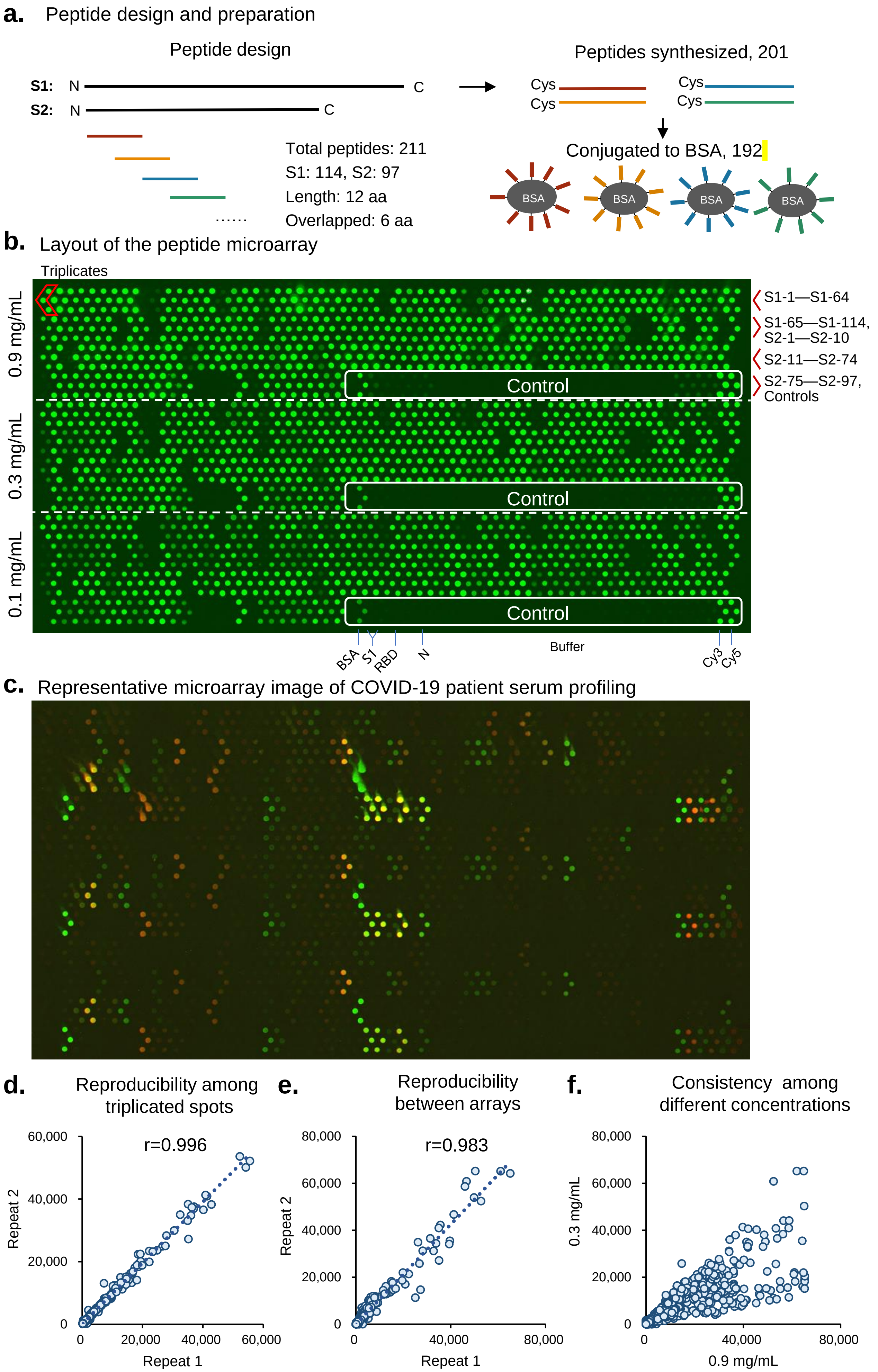

Supplementary Fig. 1. Peptide Design and microarray fabrication

Supplementary Fig. 1. Peptide design and microarray fabrication. a. Peptide design and conjugation with BSA through the cysteine on the N terminal. The numbers 201 and 192 indicates the peptides that were successfully synthesized and conjugated, respectively. b. layout of the peptide microarray. The image was from anti-BSA antibody incubation. Peptides were sequentially printed and the peptides for each row are indicated on the right. **c.** A representative merged image of one COVID-19 serum. IgG response is indicated as green, while IgM is indicated as red. **d-f.** Correlations between repeated spots of the same protein on the same array, repeats arrays and peptide groups with different concentrations.

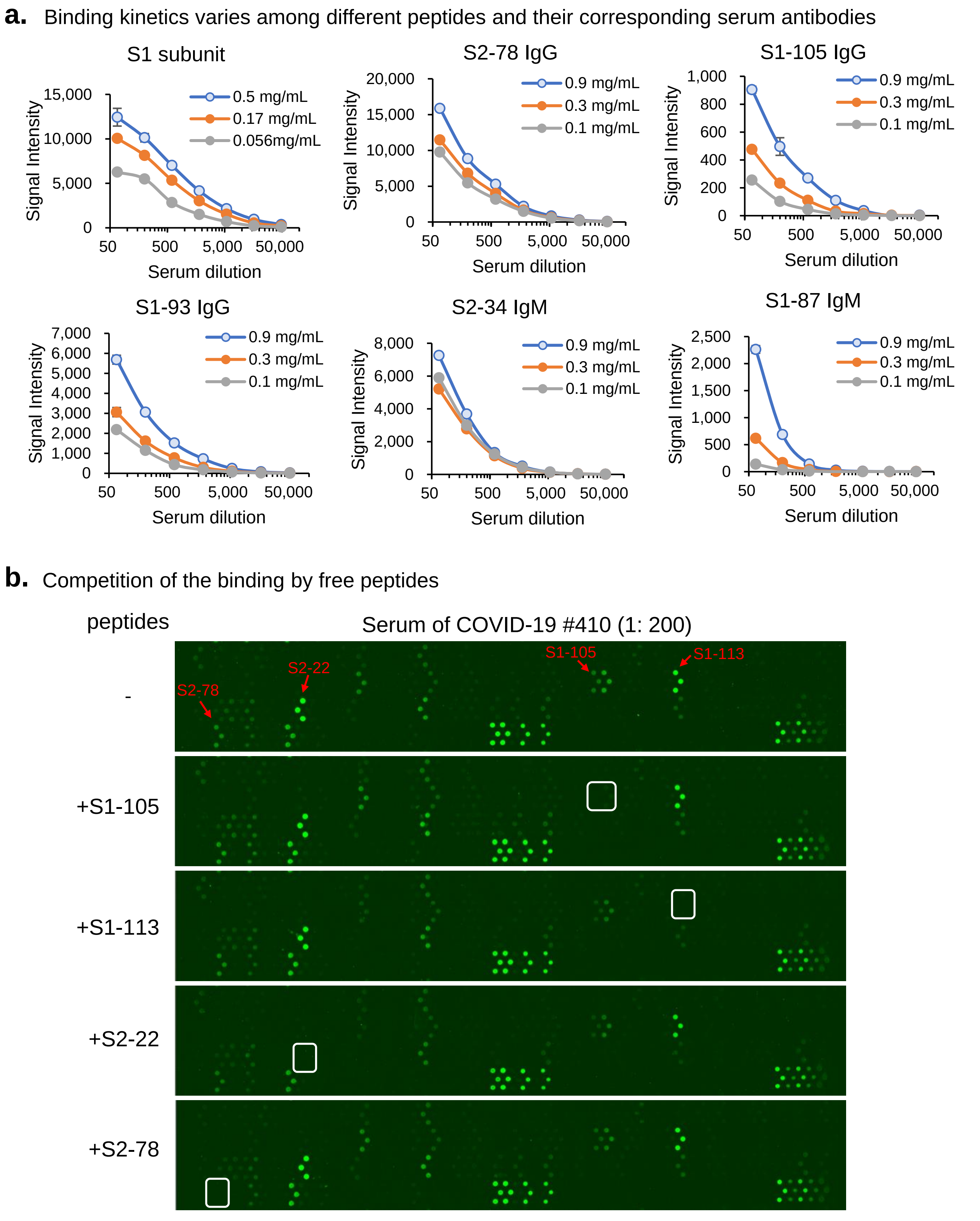

Supplementary Fig. 2. Detection of the SARS-CoV-2 specific antibody responses by using the peptide microarray. a. Dynamic change of signal intensities for some representative peptides and S1 protein in different concentrations. b. Microarray results of the competition assays with the addition of free peptides to the sera. The serum used and the dilution are labeled above. The peptides used for competition are labeled on the left and the red arrows and white rectangles indicate the position of the corresponding peptides.

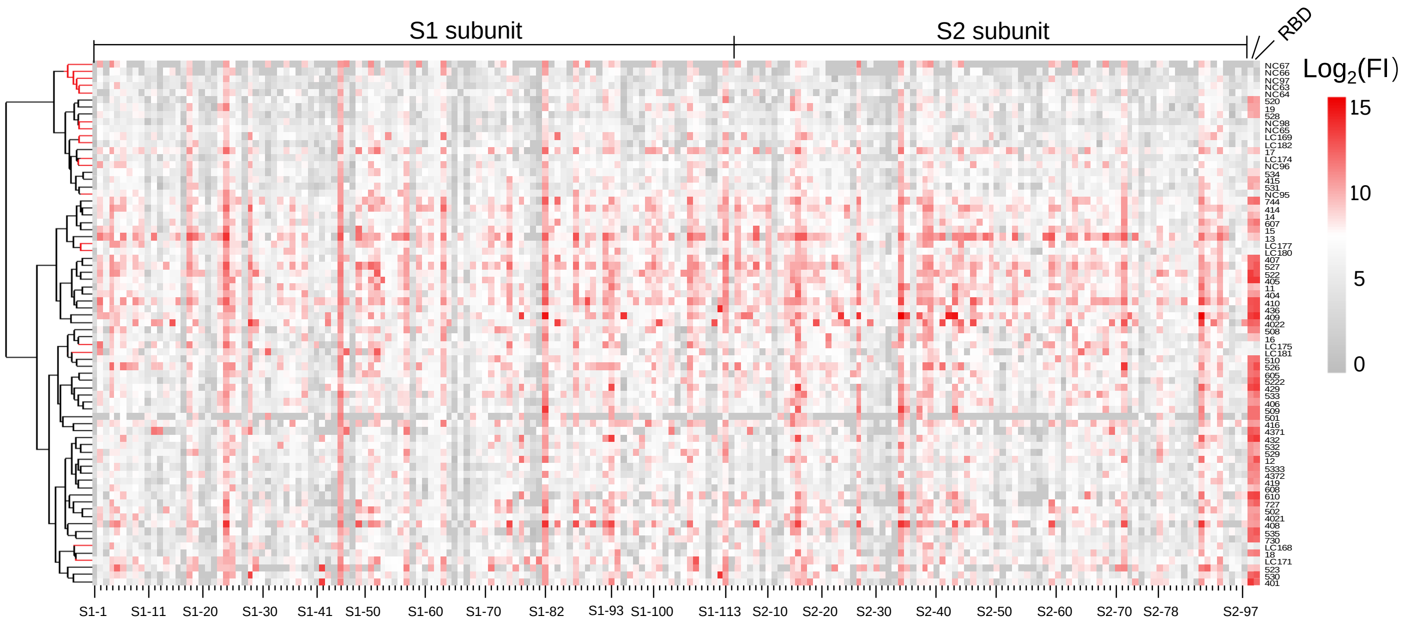

Supplementary Fig. 3. IgM responses against peptides derived from S protein. Heatmap of IgM antibody responses of 55 sera from COVID-19 convalescent patients and controls (Healthy donors and Lung cancer patients). Peptides that fully cover S protein were surveyed and S1 protein and RBD were included on the peptide microarray as controls. The peptides were sequentially arranged without clustering. FI, fluorescent intensity.

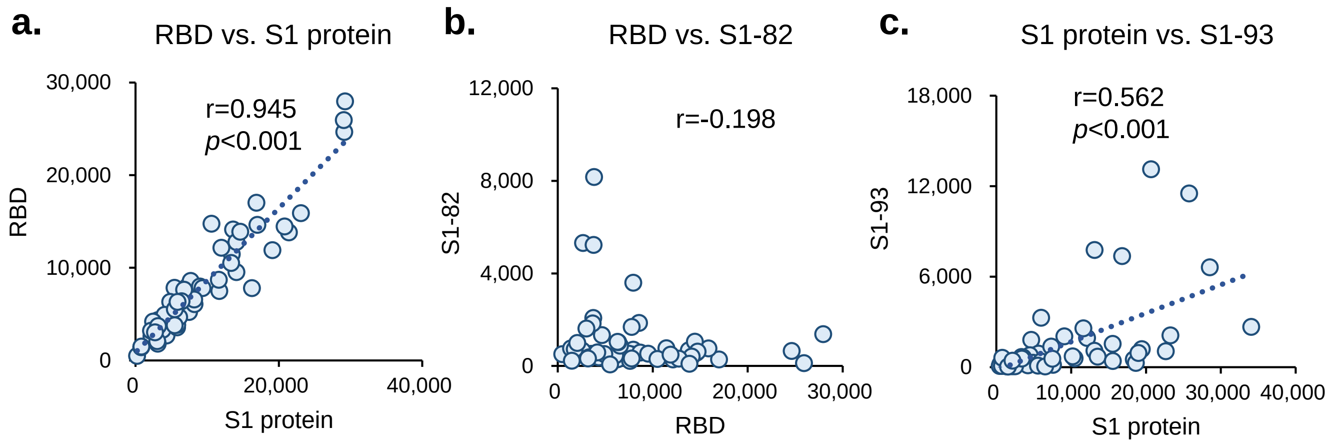

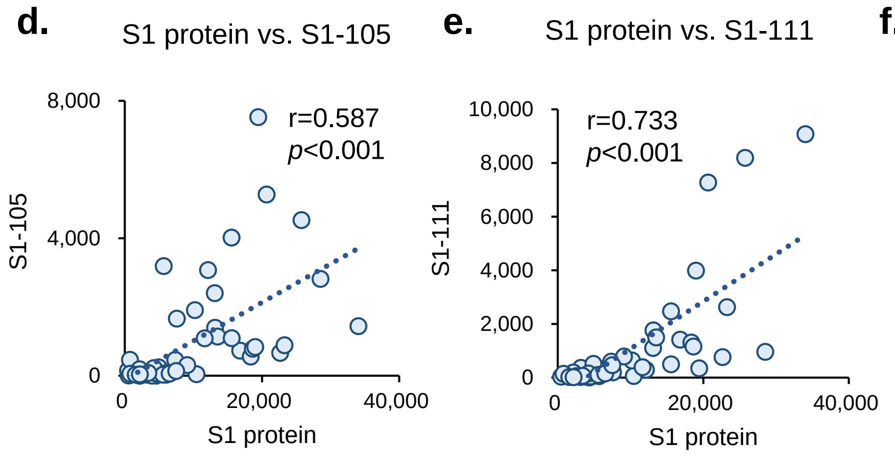

f. Correlations with S1

peptide	r	*p*
S1-93	0.562	<0.001
S1-97	0.563	<0.001
S1-100	0.108	0.437
S1-101	0.100	0.473
S1-105	0.587	<0.001
S1-106	0.392	0.003
S1-111	0.733	<0.001
S1-113	0.371	0.006

Supplementary Fig.4. Correlation analysis among antibody responses against S1 subunit derived epitopes and S1 protein. a-e. Correlations of IgG responses between two peptides, proteins or peptide and proteins. **f.** Summary of the correlations of IgG responses between representative peptides and S1 protein.

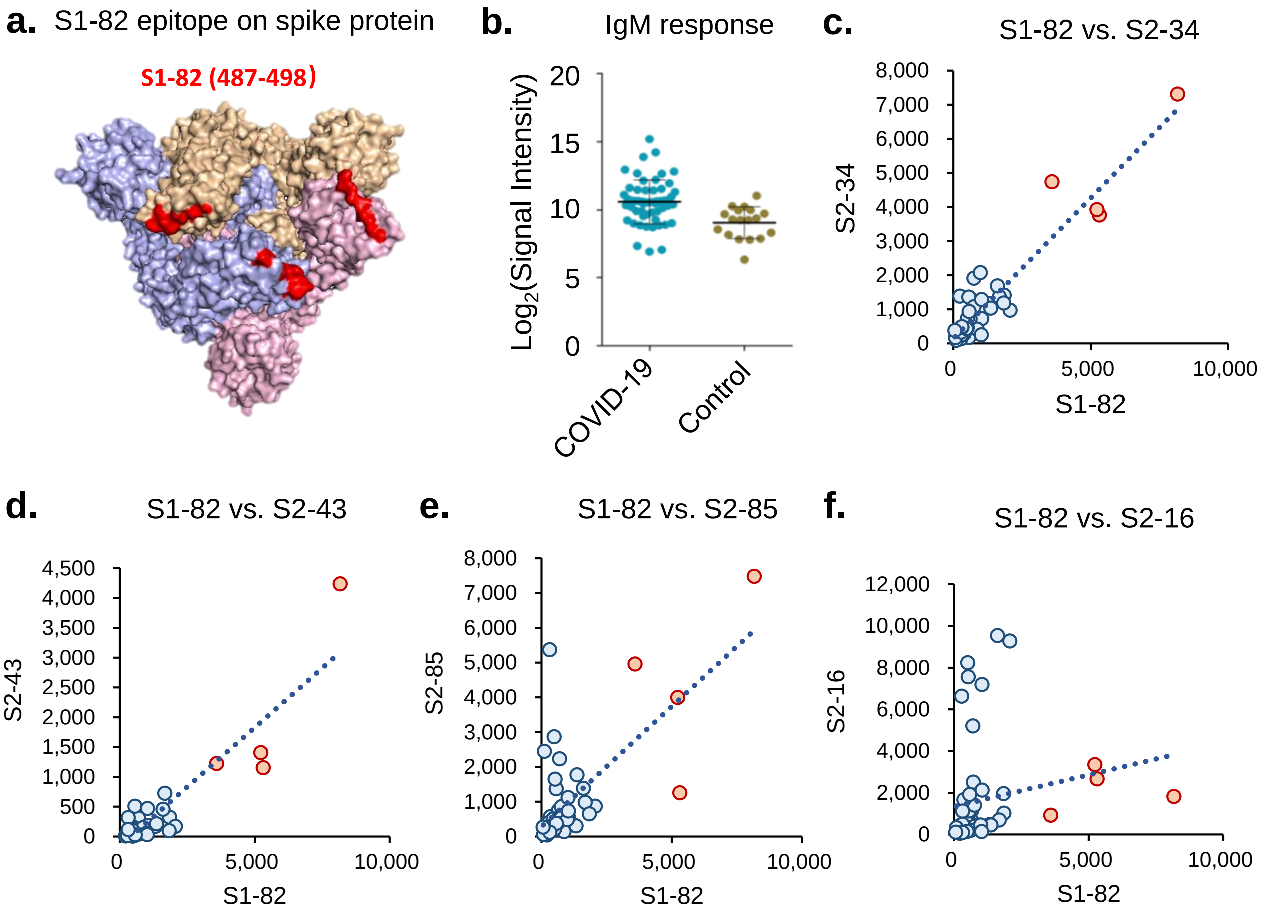

g. Sequence similarity to S1-82

S1-82	487 NCYFPLQSYGFQ 498
S2-34	884 SGWTFGAGAALQ 895
S2-43	938 LSSTASALGKLQ 949
S2-85	1190 AKNLNESLIDLQ 1201
S2-16	776 KNTQEVFAQVKQ 787

Supplementary Fig.5. Cross-activity of anti-S2-82 antibodies. a. The structural position of S1-82 on S protein (PDB: 6xyb)**. b.** IgM responses of S1-82 in COVID-19 patients (blue) and controls (yellow). c-f. correlations of IgG responses between S1-82 and other peptides. The red spots were samples with high IgG signals for S1-82. g. Sequence similarity among the indicated peptides with S1-82.

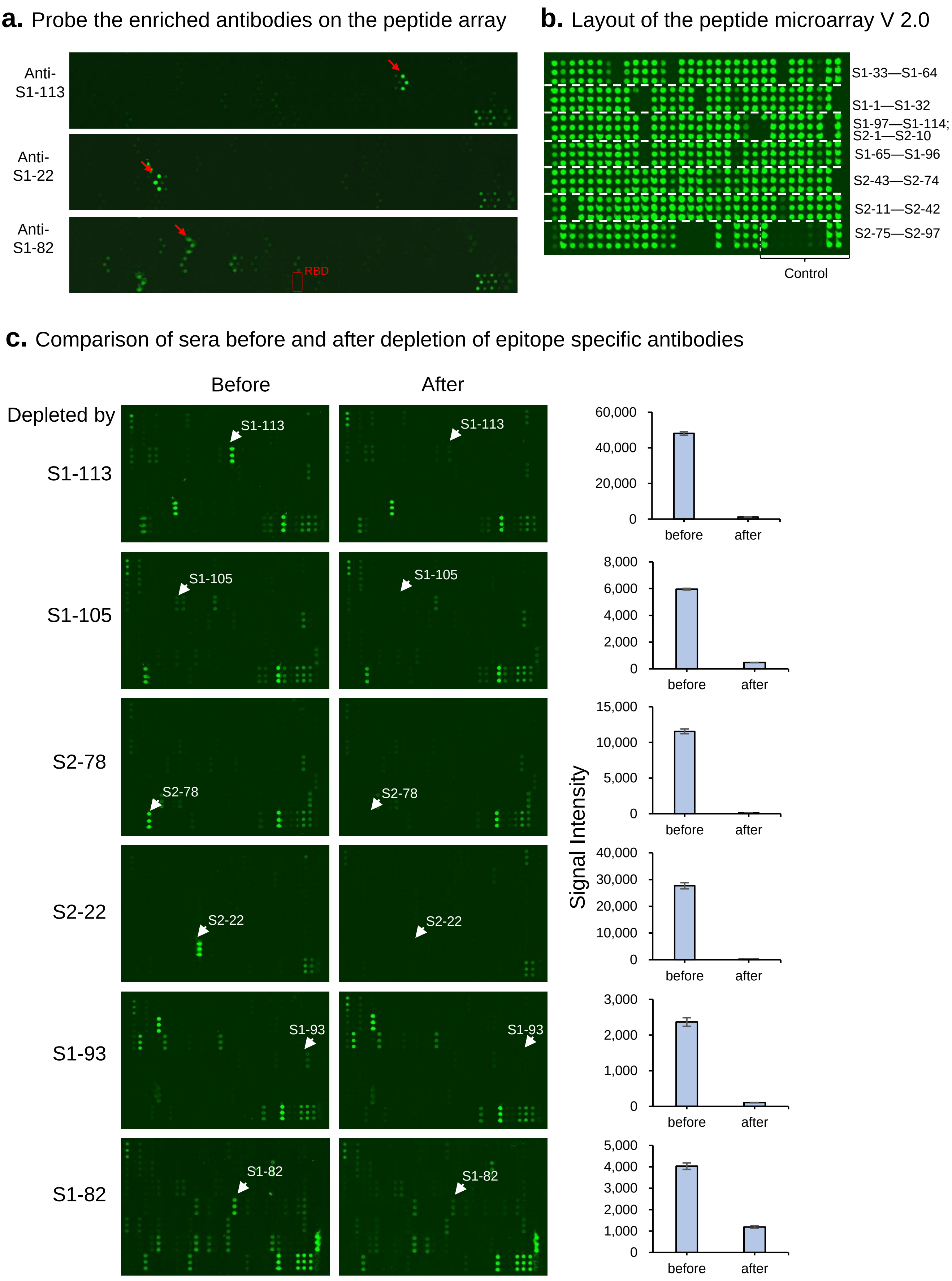
a. Probe the enriched antibodies on the peptide array
Anti-S1-113
Anti-S1-22
Anti-S1-82
RBD
b. Layout of the peptide microarray V 2.0
S1-33—S1-64
S1-1—S1-32
S1-97—S1-114; S2-1—S2-10
S1-65—S1-96
S2-43—S2-74
S2-11—S2-42
S2-75—S2-97
Control
c. Comparison of sera before and after depletion of epitope specific antibodies
Before
After
Depleted by
S1-113
S1-105
S2-78
S2-22
S1-93
S1-82
Signal Intensity
60,000
40,000
20,000
0
8,000
6,000
4,000
2,000
15,000
10,000
5,000
30,000
3,000
1,000
5,000
before
after

Supplementary Fig.6. Epitope-specific antibody depletion from sera. a. Peptide microarray results for epitope-specific antibodies. Red arrows indicate the corresponding peptides. b. Layout of the new version of the peptide microarray with 0.3 mg/mL peptides printed. c. Representative images (left) and results (right) for comparison of sera between before and after depletion of epitope specific antibodies. The positions of the corresponding peptides labeled on the left that were used for depletion are indicated by arrows.

Table S1. The peptides synthesized in this study

NO.	Peptide ID	Sart Position	Amino acid sequence	End Position	Note
1	S1-1	1	MFVFLVLLPLVS	12	
2	S1-2	7	LLPLVSSQCVNL	18	
3	S1-3	13	SQCVNLTTRTQL	24	
4	S1-4	19	TTRTQLPPAYTN	30	
5	S1-5	25	PPAYTNSFTRGV	36	
6	S1-6	31	SFTRGVYYPDKV	42	
7	S1-7	37	YYPDKVFRSSVL	48	
8	S1-8	43	FRSSVLHSTQDL	54	
9	S1-9	49	HSTQDLFLPFFS	60	
10	S1-10	55	FLPFFSNVTWFH	66	Insoluble
11	S1-11	61	NVTWFHAIHVSG	72	
12	S1-12	67	AIHVSGTNGTKR	78	
13	S1-13	73	TNGTKRFDNPVL	84	
14	S1-14	79	FDNPVLPFNDGV	90	
15	S1-15	85	PFNDGVYFASTE	96	
16	S1-16	91	YFASTEKSNIIR	102	
17	S1-17	97	KSNIIRGWIFGT	108	
18	S1-18	103	GWIFGTTLDSKT	114	
19	S1-19	109	TLDSKTQSLLIV	120	
20	S1-20	115	QSLLIVNNATNV	126	
21	S1-21	121	NNATNVVIKVCE	132	
22	S1-22	127	VIKVCEFQFCND	138	
23	S1-23	133	FQFCNDPFLGVY	144	
24	S1-24	139	PFLGVYYHKNNK	150	
25	S1-25	145	YHKNNKSWMESE	156	
26	S1-26	151	SWMESEFRVYSS	162	
27	S1-27	157	FRVYSSANNCTF	168	
28	S1-28	163	ANNCTFEYVSQP	174	
29	S1-29	169	EYVSQPFLMDLE	180	
30	S1-30	175	FLMDLEGKQGNF	186	
31	S1-31	181	GKQGNFKNLREF	192	
32	S1-32	187	KNLREFVFKNID	198	Insoluble
33	S1-33	193	VFKNIDGYFKIY	204	
34	S1-34	199	GYFKIYSKHTPI	210	
35	S1-35	205	SKHTPINLVRDL	216	
36	S1-36	211	NLVRDLPQGFSA	222	
37	S1-37	217	PQGFSALEPLVD	228	
38	S1-38	223	LEPLVDLPIGIN	234	
39	S1-39	229	LPIGINITRFQT	240	
40	S1-40	235	ITRFQTLLALHR	246	Insoluble
41	S1-41	241	LLALHRSYLTPG	252	
42	S1-42	247	SYLTPGDSSSGW	258	
43	S1-43	253	DSSSGWTAGAAA	264	
44	S1-44	259	TAGAAAYYVGYL	270	
45	S1-45	265	YYVGYLQPRTFL	276	
46	S1-46	271	QPRTFLLKYNEN	282	Insoluble
47	S1-47	277	LKYNENGTITDA	288	
48	S1-48	283	GTITDAVDCALD	294	
49	S1-49	289	VDCALDPLSETK	300	
50	S1-50	295	PLSETKCTLKSF	306	
51	S1-51	301	CTLKSFTVEKGI	312	
52	S1-52	307	TVEKGIYQTSNF	318	
53	S1-53	313	YQTSNFRVQPTE	324	
54	S1-54	319	RVQPTESIVRFP	330	
55	S1-55	325	SIVRFPNITNLC	336	
56	S1-56	331	NITNLCPFGEVF	342	
57	S1-57	337	PFGEVFNATRFA	348	
58	S1-58	343	NATRFASVYAWN	354	
59	S1-59	349	SVYAWNRKRISN	360	
60	S1-60	355	RKRISNCVADYS	366	
61	S1-61	361	CVADYSVLYNSA	372	
62	S1-62	367	VLYNSASFSTFK	378	
63	S1-63	373	SFSTFKCYGVSP	384	
64	S1-64	379	CYGVSPTKLNDL	390	
65	S1-65	385	TKLNDLCFTNVY	396	
66	S1-66	391	CFTNVYADSFVI	402	
67	S1-67	397	ADSFVIRGDEVR	408	
68	S1-68	403	RGDEVRQIAPGQ	414	
69	S1-69	409	QIAPGQTGKIAD	420	
70	S1-70	415	TGKIADYNYKLP	426	
71	S1-71	421	YNYKLPDDFTGC	432	

72	S1-72	427	DDFTGCVIAWNS	438	Failure for synthesis
73	S1-73	433	VIAWNSNNLDSK	444	
74	S1-74	439	NNLDSKVGGNYN	450	
75	S1-75	445	VGGNYNYLYRLF	456	Insoluble
76	S1-76	451	YLYRLFRKSNLK	462	
77	S1-77	457	RKSNLKPFERDI	468	
78	S1-78	463	PFERDISTEIYQ	474	
79	S1-79	469	STEIYQAGSTPC	480	
80	S1-80	475	AGSTPCNGVEGF	486	
81	S1-81	481	NGVEGFNCYFPL	492	
82	S1-82	487	NCYFPLQSYGFQ	498	
83	S1-83	493	QSYGFQPTNGVG	504	
84	S1-84	499	PTNGVGYQPYRV	510	
85	S1-85	505	YQPYRVVVLSFE	516	
86	S1-86	511	VVLSFELLHAPA	522	
87	S1-87	517	LLHAPATVCGPK	528	
88	S1-88	523	TVCGPKKSTNLV	534	
89	S1-89	529	KSTNLVKNKCVN	540	
90	S1-90	535	KNKCVNFNFNGL	546	
91	S1-91	541	FNFNGLTGTGVL	552	
92	S1-92	547	TGTGVLTESNKK	558	
93	S1-93	553	TESNKKFLPFQQ	564	
94	S1-94	559	FLPFQQFGRDIA	570	
95	S1-95	565	FGRDIADTTDAV	576	
96	S1-96	571	DTTDAVRDPQTL	582	
97	S1-97	577	RDPQTLEILDIT	588	
98	S1-98	583	EILDITPCSFGG	594	
99	S1-99	589	PCSFGGVSVITP	600	
100	S1-100	595	VSVITPGTNTSN	606	
101	S1-101	601	GTNTSNQVAVLY	612	
102	S1-102	607	QVAVLYQDVNCT	618	
103	S1-103	613	QDVNCTEVPVAI	624	
104	S1-104	619	EVPVAIHADQLT	630	
105	S1-105	625	HADQLTPTWRVY	636	
106	S1-106	631	PTWRVYSTGSNV	642	
107	S1-107	637	STGSNVFQTRAG	648	Insoluble
108	S1-108	643	FQTRAGCLIGAE	654	
109	S1-109	649	CLIGAEHVNNSY	660	
110	S1-110	655	HVNNSYECDIPI	666	
111	S1-111	661	ECDIPIGAGICA	672	
112	S1-112	667	GAGICASYQTQT	678	
113	S1-113	673	SYQTQTNSPRRA	684	
114	S1-114	679	NSPRRARGGGGS	685	
115	S2-1	686	SVASQSIIAYTM	697	Insoluble
116	S2-2	692	IIAYTMSLGAEN	703	Failure for synthesis
117	S2-3	698	SLGAENSVAYSN	709	
118	S2-4	704	SVAYSNNSIAIP	715	
119	S2-5	710	NSIAIPTNFTIS	721	
120	S2-6	716	TNFTISVTTEIL	727	Failure for synthesis
121	S2-7	722	VTTEILPVSMTK	733	
122	S2-8	728	PVSMTKTSVDCT	739	
123	S2-9	734	TSVDCTMYICGD	745	Failure for synthesis
124	S2-10	740	MYICGDSTECSN	751	
125	S2-11	746	STECSNLLLQYG	757	
126	S2-12	752	LLLQYGSFCTQL	763	
127	S2-13	758	SFCTQLNRALTG	769	Insoluble
128	S2-14	764	NRALTGIAVEQD	775	
129	S2-15	770	IAVEQDKNTQEV	781	
130	S2-16	776	KNTQEVFAQVKQ	787	
131	S2-17	782	FAQVKQIYKTPP	793	
132	S2-18	788	IYKTPPIKDFGG	799	
133	S2-19	794	IKDFGGFNFSQI	805	
134	S2-20	800	FNFSQILPDPSK	811	
135	S2-21	806	LPDPSKPSKRSF	817	
136	S2-22	812	PSKRSFIEDLLF	823	
137	S2-23	818	IEDLLFNKVTLA	829	
138	S2-24	824	NKVTLADAGFIK	835	Failure for synthesis
139	S2-25	830	DAGFIKQYGDCL	841	
140	S2-26	836	QYGDCLGDIAAR	847	
141	S2-27	842	GDIAARDLICAQ	853	
142	S2-28	848	DLICAQKFNGLT	859	
143	S2-29	854	KFNGLTVLPPLL	865	
144	S2-30	860	VLPPLLTDEMIA	871	
145	S2-31	866	TDEMIAQYTSAL	877	

146	S2-32	872	QYTSALLAGTIT	883	
147	S2-33	878	LAGTITSGWTFG	889	
148	S2-34	884	SGWTFGAGAALQ	895	
149	S2-35	890	AGAALQIPFAMQ	901	
150	S2-36	896	IPFAMQMAYRFN	907	
151	S2-37	902	MAYRFNGIGVTQ	913	
152	S2-38	908	GIGVTQNVLYEN	919	
153	S2-39	914	NVLYENQKLIAN	925	
154	S2-40	920	QKLIANQFNSAI	931	
155	S2-41	926	QFNSAIGKIQDS	937	
156	S2-42	932	GKIQDSLSSTAS	943	
157	S2-43	938	LSSTASALGKLQ	949	
158	S2-44	944	ALGKLQDVVNQN	955	
159	S2-45	950	DVVNQNAQALNT	961	
160	S2-46	956	AQALNTLVKQLS	967	
161	S2-47	962	LVKQLSSNFGAI	973	
162	S2-48	968	SNFGAISSVLND	979	
163	S2-49	974	SSVLNDILSRLD	985	
164	S2-50	980	ILSRLDKVEAEV	991	
165	S2-51	986	KVEAEVQIDRLI	997	
166	S2-52	992	QIDRLITGRLQS	1003	
167	S2-53	998	TGRLQSLQTYVT	1009	
168	S2-54	1004	LQTYVTQQLIRA	1015	
169	S2-55	1010	QQLIRAAEIRAS	1021	
170	S2-56	1016	AEIRASANLAAT	1027	
171	S2-57	1022	ANLAATKMSECV	1033	
172	S2-58	1028	KMSECVLGQSKR	1039	
173	S2-59	1034	LGQSKRVDFCGK	1045	
174	S2-60	1040	VDFCGKGYHLMS	1051	
175	S2-61	1046	GYHLMSFPQSAP	1057	
176	S2-62	1052	FPQSAPHGVVFL	1063	
177	S2-63	1058	HGVVFLHVTYVP	1069	
178	S2-64	1064	HVTYVPAQEKNF	1075	
179	S2-65	1070	AQEKNFTTAPAI	1081	
180	S2-66	1076	TTAPAICHDGKA	1087	
181	S2-67	1082	CHDGKAHFPREG	1093	
182	S2-68	1088	HFPREGVFVSNG	1099	
183	S2-69	1094	VFVSNGTHWFVT	1105	
184	S2-70	1100	THWFVTQRNFYE	1111	
185	S2-71	1106	QRNFYEPQIITT	1117	
186	S2-72	1112	PQIITTDNTFVS	1123	
187	S2-73	1118	DNTFVSGNCDVV	1129	
188	S2-74	1124	GNCDVVIGIVNN	1135	Failure for synthesis
189	S2-75	1130	IGIVNNTVYDPL	1141	
190	S2-76	1136	TVYDPLQPELDS	1147	
191	S2-77	1142	QPELDSFKEELD	1153	
192	S2-78	1148	FKEELDKYFKNH	1159	
193	S2-79	1154	KYFKNHTSPDVD	1165	
194	S2-80	1160	TSPDVDLGDISG	1171	
195	S2-81	1166	LGDISGINASVV	1177	
196	S2-82	1172	INASVVNIQKEI	1183	
197	S2-83	1178	NIQKEIDRLNEV	1189	
198	S2-84	1184	DRLNEVAKNLNE	1195	
199	S2-85	1190	AKNLNESLIDLQ	1201	
200	S2-86	1196	SLIDLQELGKYE	1207	
201	S2-87	1202	ELGKYEQYIKWP	1213	
202	S2-88	1208	QYIKWPWYIWLG	1219	
203	S2-89	1214	WYIWLGFIAGLI	1225	Failure for synthesis
204	S2-90	1220	FIAGLIAIVMVT	1231	Failure for synthesis
205	S2-91	1226	AIVMVTIMLCCM	1237	Failure for synthesis
206	S2-92	1232	IMLCCMTSCCSC	1243	Failure for synthesis
207	S2-93	1238	TSCCSCLKGCCS	1249	
208	S2-94	1244	LKGCCSCGSCCK	1255	Insoluble
209	S2-95	1250	CGSCCKFDEDDS	1261	
210	S2-96	1256	FDEDDSEPVLKG	1267	
211	S2-97	1262	EPVLKGVKLHYT	1273	

Table S2. Serum samples used in this study

COVID-19 patient group		n=55
Gender	Male	27
	Female	28
Age		41.5±14.9
Severity	mild cases	8
	moderate cases	47
Days after onset		27.5±7.7
hospital stay (days)		14.0±5.6
**Control group**		**n=18**
Lung cancer patients (LC)		9
Health control (HC)		9
Gender	Male	8
	Female	10
Age		50.4±12.5
Sample collection (year)		2017-2018
